# Open surgical approach for infected mesenteric pseudocyst presenting as lifelong, migratory abdominal pain—A case report

**DOI:** 10.1016/j.ijscr.2019.10.041

**Published:** 2019-10-29

**Authors:** Thomas Serena, Raisa Gao, Kelly Dinnan

**Affiliations:** Beaumont Health Farmington Hills, Department of General Surgery, 28050 Grand River Avenue, Farmington Hills, MI, 48336, USA

**Keywords:** Mesenteric cyst, Case report, Intestinal pseudocyst

## Abstract

**Introduction:**

Mesenteric psuedocysts are rare tumors of the gastrointestinal mesentery that are seldom symptomatic. Although these benign tumors are most commonly found incidentally during work-up for other pathology, they can be troublesome in select patients based off size, location and risk of malignant transformation. This case is reported in accordance with SCARE Criteria [1].

**Presentation of case:**

A 24 year-old-male presents with life-long migratory abdominal pain presents with a one week history of acute pain associated with nausea. Computed tomography revealed free fluid in the pelvis and a thin-walled mesenteric cyst within the left, mid-abdominal mesentery measuring approximately 4.3 × 4.0 × 4.0 cm. The patient was admitted for resuscitation and planned delayed operative intervention.

**Discussion:**

The patient underwent complete open enucleation secondary to location and in an attempt to limit injuries to or resection of small bowel. Pathological analysis revealed a mesenteric cyst with fluid culture positive for *Propionibacterium acnes* without true cystic wall consistent with an infected mesenteric pseudocyst. These lesions are difficult to diagnose secondary to varied presentation and lack of pathognomonic clinical, laboratory and imaging findings. Mesenteric pseudocyst have a low rate of recurrence after removal; however, surgical management is mandated due to risks of malignant transformation.

**Conclusion:**

This is a rare case of a mesenteric pseudocyst of small size presenting with lifelong abdominal pain secondary to its location near the root of the mesentery and inflammatory reaction secondary to infection. It is important to maintain a high index of suspicion for mesenteric cyst as many complications may result if misdiagnosed or without proper surgical management.

## Highlights

• Mesenteric cysts are rare tumors of the gastrointestinal mesentery that are seldom symptomatic, often found incidentally.
• This is a rare case of a small mesenteric cyst presenting with abdominal pain secondary to intimate connection to the root of the mesentery.
• Including this study, multiple case reports and case series have been performed without consensus for best modality of treatment.
• This literature would benefit from a larger prospective study delineating treatment guidelines.

## 1 Introduction

Mesenteric Psueodycst are rare type of mesenteric cyst, found in less than 1 out of 250,000 hospital admissions [2]. These benign neoplasms can be anywhere along the mesentery from the duodenum to rectum, the majority being asymptomatic and found incidentally on imaging or during an unrelated surgery [4]. These lesions are difficult to diagnose secondary to varied presentation and lack of pathognomonic clinical, laboratory and imaging findings. This case report examines a small mesenteric cyst presenting in a 24-year-old male with reported lifelong migratory abdominal pain and nausea for one week prior to admission. After making the initial diagnosis on CT, successful surgical management with open laparotomy lead to the complete enucleation of the cyst and resolution of the patient’s symptoms. These lesions have a low rate of recurrence after removal; however, surgical cure is mandated due to risks of malignant transformation. The case was diagnosed by clinical examination and computed tomography and then managed with open laparotomy at a community-based hospital. This case demonstrates the importance of consider these pathologic lesions due to the morbidity if misdiagnosed or without proper management. This case is reported in accordance with SCARE Criteria.

## 2 Presentation of case

A 24-year-old Caucasian male presented to the emergency department for unremitting left lower abdominal pain for one week that intensified over the last 24 h with nausea. The patient reported a lifelong history of abdominal pain as mild, self-limiting episodes. Past medical history was significant for dilated cardiomyopathy diagnosed 5 years prior to presentation. Past surgical and family history was noncontributory.

**Fig. 1:**
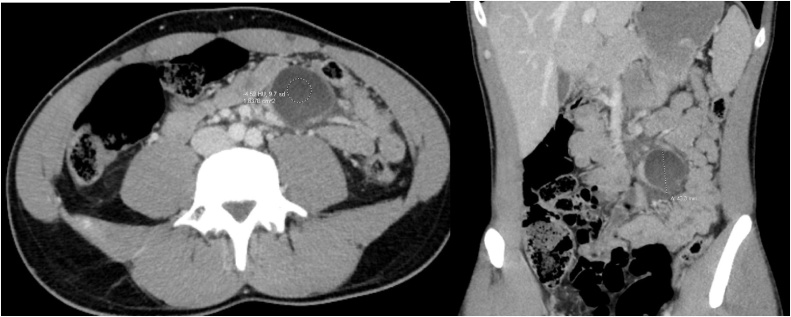
Computed Tomography of abdomen and Pelvic with IV contrast demonstrating left mid-abdominal mesenteric fluid collection near root of mesentery measuring up to 4.3 cm in largest dimension.

On clinical exam, all vitals were found to be within normal parameters. The patient’s abdomen was soft, non-distended and tender to palpation in the left abdomen lateral to the umbilicus. No masses, organomegaly, nor hernias were palpated. Laboratory testing was significant for a leukocytosis of 12.4 bil/L and neutrophil count of 10.4 bil/L. Computed tomography revealed free fluid in the pelvis with a thin-walled unilocular cystic structure within the left, mid-abdominal mesentery adjacent to multiple small bowel loops and numerous vessels, measuring 4.3 × 4.0 × 4.0 cm in craniocaudal, AP and transverse dimension. The cystic structure measured Hounsfield units of -4.59 consistent with simple fluid. Differential diagnosis included mesenteric cyst, omental cyst, enteric duplication cyst, lymphoma, or infectious etiology (Fig. 1).

Conservative management was attempted based on the patient’s HPI, clinical presentation and imaging. Decision was made to move forward with surgical intervention secondary to progression of the patient’s symptoms. Pre-intervention discussions centered on surgical approach, with consideration for excision of cyst, enucleation, bowel resection, laparoscopic resection, or endoscopic resection. In reflection of the size and location of the mass and surgical yield, decision was made to attempt an open surgical approach.

**Fig. 2:**
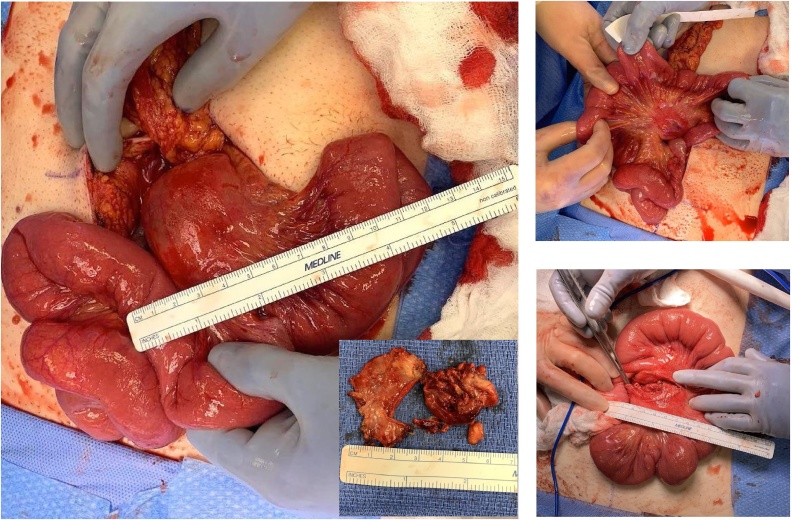
Intraoperative mobilization of cyst at root of mesentery. Enucleation of cyst performed with tissue gross pathology pictured.

The patient was consented and underwent exploratory laparotomy by a team of experienced surgeons and residents. A mass was found intraoperatvely, initially unable to be delivered through the incision secondary to location and adherent nature to the root of the mesentery. The cyst was eventually mobilized and carefully dissected from surrounding tissue. Secondary to tightly embedded nature within the mesentery and with surrounding intestine, the cyst was unavoidably entered producing thick white drainage then collected and sent to pathology. Decision was then made to perform complete enucleation of the cyst with ligation of all feeding vessels. No resection of bowel was found to be necessary (Fig. 2).

The postoperative period was uneventful and the patient was discharged without complication on postoperative day two. He was seen in office for follow up one week later and with complete resolution of previous symptoms. The patient has no need for further follow-up examinations.

**Fig. 3:**
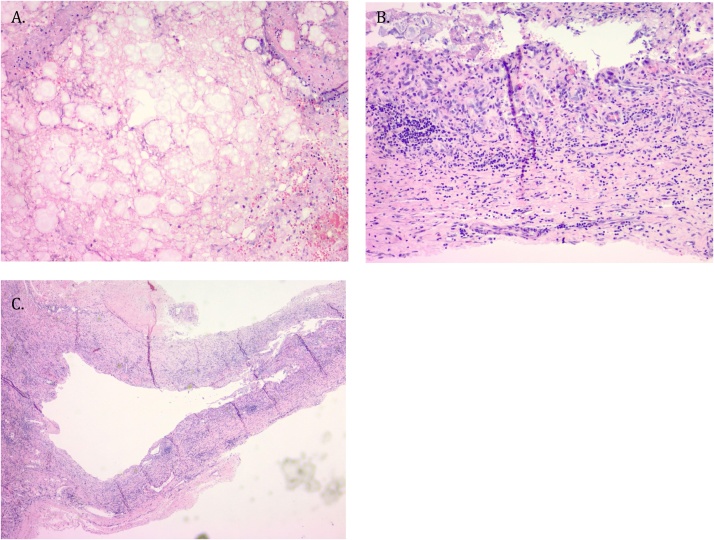
A. 2× magnification Demonstration of cyst wall without definitive lining. B. 10 x magnification of fragments of nodular cystic fat necrosis, sheets of membranous tissue with adherent yellow-tan fat and an aggregate of red-gray soft grumous material. C 10 x magnification of fat necrosis with inflamed fibrous wall without definitive lining. Central portion of fat necrosis with inflamed tissue.

Surgical pathology tissue showed fragments of nodular cystic fat necrosis consistent with mesenteric cyst. Fluid cytology was negative for malignancy. Fluid culture grew *Propionibacterium acnes*, an opportunistic, gram-positive pathogen found in the normal skin flora, primarily known for its role in acne (Fig. 3).

## 3 Discussion

Mesenteric cysts are rare intra-abdominal tumors with current belief they arrive from failure in communications between lymphatic or venous systems within the gastrointestinal tract. They may extend from the base of the mesentery into the retroperitoneum and can be found anywhere in the mesentery of the gastrointestinal tract from the duodenum to the rectum [7]. Mesenteric cysts are most commonly found in the small bowel mesentery. The large bowel mesentery is the second most common location followed by the retroperitoneum.^2^ Characteristics vary between single to multiple and simple, unilocular or multilocular, containing hemorrhagic, chylous, serous or infected fluid [8,9]. In 1950, Beahrs et al. classified mesenteric cysts into four categories: (1) embryonic and developmental cysts, (2) traumatic cysts, (3) neoplastic cysts, and (5) infective and degenerative cysts [10]. More recently, de Perrot et al. suggested an updated system of classification based on histopathological features: 1) cysts of lymphatic origin (simple lymphatic cyst and lymphangioma), (2) cysts of mesothelial origin (simple mesothelial cyst, benign cystic mesothelioma, and malignant cystic mesothelioma), (3) cysts of enteric origin (enteric cyst and enteric duplication cyst), (4) cysts of urogenital origin, (5) mature cystic teratoma (dermoid cysts), and (6) pseudocysts (infectious and traumatic cysts) [11]. In this classification the patient would most likely be classified as a pseudocyst of likely infectious etiology.

Mesenteric cyst will most commonly present one of three ways: chronic pain, asymptomatic, or as an acute abdomen. Patients may report chronic nonspecific diffuse abdominal pain postulated to be secondary to stretching of the mesentery and peritoneum by the cyst or vascular compromise to the bowel. Symptoms of intermittent colicky pain, nausea, vomiting, constipation and diarrhea have been reported [5]. Morbidity dependent on size and location of the cysts. Acute cases are usually secondary to obstruction, rupture, hemorrhage into the cyst, or infection of abscess of the cyst. These episodes have been reported to clinically present similar to appendicitis [5] and even abdominal aortic aneurysm [12].

Ultrasound and CT are beneficial in diagnosis with the ability to distinguish between solid and cystic components. Contrast CT provides further information regarding relation to vascular structures [6]. One study showed the utility of chemical shift MRI in distinguishing the origin of the cyst by detecting its lipid content thereby further guiding treatment [13]. Laboratory results tend to be nonspecific and do not offer a clear diagnosis for mesenteric cyst [4].

Surgical excision is recommended due to potential for malignant transformation (<3 %), obstruction or recurrence. Preferred treatment is complete enucleation, associated with a high cure rate. Excision with enterectomy may be necessary due to intimacy with bowel or vessels feeding the bowel [3]. Marsupialization can be performed if the previous methods are not possible, however, is not preferred based on higher rates of recurrence [4,14]. Partial excision or drainage of cysts are deemed unacceptable treatment options for similar reasons [3].

In this case, excision of the cyst would compromise a significant length of bowel secondary to its intimate relation with the mesenteric root vasculature. Many successful laparoscopic cases have been documented. There has yet to be a large study to evaluate the effectiveness or best approach to the technique. Advantages to laparoscopy include decreased postoperative pain, shorter hospital stay, earlier return to normal activity and smaller incision. In many cases, complete excision was achieved without recurrence of the cyst [[15], [16], [17]]. In the appropriately selected patient, a laparoscopic approach can be considered. Open approach was utilized in this patient secondary to the likely difficulty and risks of attempting this procedure laparoscopically due to location and small size of this lesion.

Fluid culture grew *Propionibacterium acnes*, an opportunistic gram-positive pathogen found in normal skin flora, primarily known for its role in acne. Reports have also shown that this bacterium is responsible for many cases of postoperative infections and other chronic infections. Reports of *P. acnes* causing endocarditis of prosthetic and native heart valves, corneal infections, postoperative endophthalmitis, focal intracranial infections and cerebral spinal fluid shunt infections are published [18]. Studies have shown *P. acnes* isolated in cases of chronic inflammatory disease including chronic prostatitis leading to prostate cancer, chronic recurrent multifocal osteomyelitis, sarcoidosis and sciatica [19]. The bacterium has been found in gastric mucosa of healthy individuals [20]. This case report to be the first time *Propionibacterium acnes* has been isolated in a mesenteric pseudocyst.

## 4 Conclusion

Mesenteric cysts are rare tumors seldomly included in the differential diagnosis of a patient presenting with chronic or acute abdominal pain. These lesions are difficult to diagnose secondary to varied presentation and lack of pathognomonic clinical, laboratory and imaging findings. It is important to consider these pathologic lesions due to the morbidity if misdiagnosed or without proper management. These lesions have a low rate of recurrence after removal; however, surgical cure is mandated due to risks of malignant transformation. This case report examines a small mesenteric cyst presenting in a 24-year-old male with reported lifelong migratory abdominal pain and nausea for one week prior to admission. After making the initial diagnosis on CT, successful surgical management with open laparotomy lead to the complete enucleation of the cyst and resolution of the patient’s symptoms.

## Funding

None.

## Ethical approval

IRB Approval exemption has been given from the IRB/Resident Review committee.

## Consent

Consent obtained in person with patient, consent form signed by patient and two physicians.

## Author contribution

Thomas J Serena, D.O. M.A.: Thomas.Serena@beauont.org, Primary author, corresponding author, study concept/design, analysis and interpretation.

Kelly Dinnan, D.O.: Kelly.Dinnan@beaumont.org: Guarantor, Contributing Author.

Raisa Gao: Gaoraisa@msu.edu, Contributing Author, review.

## Registration of research studies

Retrospective review of case without identification or new/investigational treatments.

Registered with Research Registry: researchregistry5170.

## Guarantor

Kelly Dinnan, DO.

## Provenance and peer review

Not commissioned, externally peer-reviewed.

## Declaration of Competing Interest

None.
